# Roles of MYC-targeting long non-coding RNA MINCR in cell cycle regulation and apoptosis in non-small cell lung Cancer

**DOI:** 10.1186/s12931-019-1174-z

**Published:** 2019-09-03

**Authors:** Shengjie Chen, Tianyi Gu, Ziwen Lu, Lipeng Qiu, Guoliang Xiao, Xiaozhong Zhu, Feng Li, Hui Yu, Gang Li, Hanqing Liu

**Affiliations:** 1 Department of Cardiothoracic Surgery, Affiliated Hospital of Jiangsu University, Zhenjiang, 212001 Jiangsu Province China; 2 School of Medicine, Jiangsu University, Zhenjiang, 212013 Jiangsu Province China; 3 School of Pharmacy, Jiangsu University, Zhenjiang, 212013 Jiangsu Province China; 4 Institute of Life Sciences, Jiangsu University, Zhenjiang, 212013 Jiangsu Province China

**Keywords:** Long non-coding RNA, MINCR, Non-small cell lung cancer, C-Myc

## Abstract

**Background:**

Non-small cell lung cancer (NSCLC) is one of the leading causes of cancer death in the world, and has a relatively low survival rate. Long non-coding RNAs (lncRNAs) have been demonstrated to modulate cancer progression through a variety of molecular mechanisms. We sought to investigate the role and potential mechanism of MYC-induced long non-coding RNA (MINCR) in NSCLC.

**Methods:**

Expression levels of MINCR was first identified using The Cancer Genome Atlas (TCGA), further confirmed with specimens from 29 NSCLC patients and three cell lines using qRT-PCR. Overexpression and knockdown of MINCR were performed in NSCLC cell lines through MINCR overexpression vectors and synthesized siRNAs, respectively. The roles of MINCR in NSCLC cell lines, such as cell proliferation, cell cycle arrest, and apoptosis, were identified by MTT, flow cytometry, and Western blot. The modulation of MINCR-regulated genes, including c-Myc and its downstream effectors, as well as apoptosis-associated genes, was analyzed using Western blot.

**Results:**

MINCR expression was increased in NSCLC patients from TCGA datasets, and was also significantly increased in our collected specimens from NSCLC patients and NSCLC cell lines. Knocking down of MINCR greatly inhibited the growth of NSCLC cell lines PC9 and A549. In addition, silencing of MINCR induced cell cycle arrest and apoptosis. Furthermore, silencing of MINCR reduced the expression levels of oncogene c-Myc and its downstream cyclin A, cyclin D, CD4, and CDK2, as well as apoptosis-associated Bcl-2, while significantly increased the expression levels of cleaved PARP-1. In the meantime, overexpression of MINCR remarkably enhanced cell proliferation of PC9 cells and activated c-Myc and its downstream effectors.

**Conclusion:**

MINCR exerted inhibitory effects on the cell cycle arrest and apoptosis of NSCLC cells by activating c-Myc and its downstream effectors, suggesting that this lncRNA could be used as a potential therapeutic target for the treatment of NSCLC.

**Electronic supplementary material:**

The online version of this article (10.1186/s12931-019-1174-z) contains supplementary material, which is available to authorized users.

## Introduction

Lung cancer is one of the leading causes of malignancy induced human death. Non-small cell lung cancer (NSCLC) is a major type of lung cancer, accounting for 80% of all cases of lung cancers. Despite some effective progresses has been made in chemotherapy and targeted molecular therapies, the 5-year survival rate of lung cancer remains low, ranging from 10 to 30% all over the world. Thus, it is critically important to elucidate the underlying molecular mechanisms of NSCLC to develop noval therapeutic drugs.

Over the past decade, the development in deep sequencing of mammalian transcriptomes has led to the discovery of more than 100,000 non-coding RNAs [1, 2]. Sharing certain structural similarities with protein-coding mRNAs, long non-coding RNAs (lncRNAs) refer to transcripts that are longer than 200 nucleotides but without protein-coding potential [2–4]. It has been revealed that lncRNAs are very heterogeneous in their mechanisms of function. Therefore, without any surprise, as the researches go on, lncRNAs have been demonstrated to exhibit versatile functions in diverse biological processes [5–8]. More importantly, recent studies showed that lncRNAs are involved in tumorigenesis and development of many kinds of cancers [9–12].

About 3 years ago, Doose et al. discovered that MYC-induced lncRNA (MINCR) was able to modulate the transcriptional network of MYC (c-Myc) in Burkitt lymphoma cells [13]. After that, MINCR was found to be significantly increased, and play an oncogenic role in cancers, such as gallbladder cancer and hepatocellular carcinoma [14, 15]. Wang et al. revealed that MINCR promotes gallbladder cancer progression in part by sponging miR-26a-5p and activating enhancer of zeste homolog 2 (EZH2) signaling; while Cao et al. reported that MINCR enhances the proliferation, migration, and invasion of hepatocellular carcinoma cells [14, 15]. All these studies imply that MINCR could be a therapeutic target as well as prognostic marker for cancer treatment.

As we are interested in the treatment of NSCLC, we screened a panel of lncRNAs, and found that MINCR was highly expressed in patient samples and cell lines of NSCLC. In the current study, we evaluated the function of MINCR in the proliferation and apoptosis of NSCLC cell lines in vitro, and then investigated the impact of MINCR on oncogene c-Myc and its downstream effectors, as well as apoptosis-associated genes to reveal the underlying mechanism beneath these phenomena.

## Materials and methods

### Data collection from the Cancer genome atlas (TCGA)

The expression of MINCR in two subtypes of NSCLC, including lung adenocarcinoma (LUAD) and lung squamous carcinoma (LUSC), were extracted from TCGA. For LUAD, The expression of MINCR was collected from 526 tumor and 59 non-tumor samples; for LUSC, the expression of MINCR was collected from 501 tumor and 49 non-tumor samples.

### Specimens and ethics statement

Twenty-nine new NSCLC patients who have not received any anti-cancer radio- or chemotherapy were enrolled in this study. After surgical processes in Affiliated Hospital of Jiangsu university, their paired tissue specimens (NSCLC tissue and para-tumor tissue) were immediately preserved in RNA-fixer reagent (Bioteke, Beijing, China), and frozen at − 80ºC until use. All these specimens were pathologically confirmed by two experienced pathologists. Clinicopathological characteristics of these NSCLC patients are summarized in Table 1.

**Table 1:** Clinicopathological characteristics of NSCLC patients

Characteristics	Number (*n* = 29)	Percent (%)
Age (years)		
<60	10	34.5
≥60	19	65.5
Gender		
Male	17	58.6
Female	12	41.4
History of Smoking		
Never	18	62.1
Current	11	37.9
Tumor size (cm)		
3 cm or less	16	55.2
over 3 cm	13	44.8
Histological grade		
High	3	10.3
High to middle	3	10.3
Middle	5	17.2
Middle to low	6	20.7
Low	2	6.9
Other	10	34.5
Histological classification		
Adenocarcinoma	21	72.4
Squamous cell carcinoma	6	20.7
Adenosquamous carcinoma	2	6.9
Lymph node metastasis		
Yes	5	17.2
No	24	82.8

This study was approved by the Human Research Ethics Committee of Jiangsu University. All the subjects have signed informed consent before the surgery.

### Antibodies and reagents

The antibodies for the Western blot were c-Myc (1:1000, Cell Signaling Technology), cylin-dependent kinase (CDK) 4 (1:500, Santa Cruz), CDK2 (1:500, Santa Cruz), Cyclin A (1:1000, Santa Cruz), Cyclin D (1:1000, Cell Signaling Technology), β-actin (1:1000, BEIDI Bio.). Other reagents: TRIzol Reagent (Life Technologies, Grand Island, NY, USA), V-FITC/PI staining reagent (Yeasen Biotech, Shanghai, China), BCA Protein Assay Kit (Beyotime Biotech, Nantong, China), and MTT (3-[4]-2, 5-diphenyltetrazolium bromide thiazolyl blue) (Sigma-Aldrich, St Louis, MO, USA).

### Cell culture

Human non-small cell lung cancer cell lines (PC9, HCC827, A549) and human normal lung cell line 16HBE were obtained from the American Type Culture Collection (ATCC, USA). PC9 and HCC827 cells were grown in RPMI1640 (Gibco, Thermo Fisher Scientific, USA); while A549 and 16HBE cells were grown in Dulbecco’s modified Eagle’s medium (DMEM). Both media were supplemented with 10% FBS, 2 mmol/L L-glutamine and 100 U/mL penicillin/100 μg/mL streptomycin (Life Technologies, Grand Island, NY, USA) in a humidified 37 °C incubator with 5% CO_2_.

### Cell transfection

Two small interfering RNAs (siRNAs) against MINCR, si-MINCR-01 and si-MINCR-02, and negative control siRNA (si-NC) were purchased from Shanghai Gene Pharma Company (China). The siRNA sequences used were as follows: si-MINCR-01 (forward: 5′-GAGCCUUGUUUGCCAUUAATT-3′; reverse: 5′-UUAAUGGCAAACAAGGCUCTT-3′), si-MINCR-02 (forward: 5′-GGGAAGAGUGCGUCUGUGATT-3′; reverse: 5′-UCACAGACGCACUCUUCCCTT-3′), and si-NC (forward: 5′- CGUACGCGGAAUACUUCGATT-3′; reverse: 5′- UCGAAGUAUUCCGCGUACGTT-3′). For over-expression studies, the full-length MINCR was synthesized by GeneScript Co. (Nanjing, China), and then cloned into the BamHI and EcoRI sites of pcDNA 3.1(+) expression vector. Transfection was carried out in accordance with the instructions of Lipofectamine 2000 transfection reagent (Invitrogen) [15–17]. The final concentration of MINCR siRNAs and their negative controls for transfection were 25 nM.

### Cell viability assay

Cells were cultured in a 96-well plate with 3000 cells per well. Silencing the MINCR to measure proliferation requires siRNA reverse transfection while seeding cells. Cell viability was measured every 24 h thereafter for 72 h, and cell viability was measured by the MTT method according to the manufacturer’s instructions. Each experiment was repeated independently at least three times [13, 18, 19].

### Immunofluorescence assay

Immunofluorescence staining was carried out using the method described in several previous studies [19–21]. 72 h post transfection, cells were fixed in 4% paraformaldehyde (Sigma-Aldrich, St Louis, MO, USA) for 10 min and permeabilized by 0.2% Triton X-100 for 5 min at room temperature. Fixed cells were washed three times in PBS and blocked for 30 min with 3% BSA in PBS and then incubated with an anti-Ki-67 (Santa Cruz, Dallas, TE, USA) antibody for 2 h at RT, washed three times in PBS and incubated with goat anti-mouse Alexa-fluor 594 secondary antibody (Thermo Scientific, Waltham, MA, USA) for 1 h at room temperature. Finally, cells were incubated with 4′,6-diamidino-2-phenylindole (DAPI) for 5 min at room temperature. Images were captured using Nikon Eclipse.

### Western blot analysis

The standard procedures for Western blotting experiments in this study were performed according to the procedures given in previous studies [12, 18, 22, 23]. Cells were collected 72 h post transfection, and lysed in Lysis Bufer (50 mM Tris-HCl (pH 7.4), 150 mM NaCl, 1 mM PMSF, 1 mM EDTA, 1% Triton x-100, 1% Halt Phosphatase Inhibitor Cocktail EDTA-free 100× (Thermo Scientific, Rockford, IL, USA), and 30 μg of protein was run over 10% or 12% SDS-PAGE gels and eletrophoretically transferred to a PVDF membrane (immobilon-PSQ Transfer Membrace, Millpore, Billerica, MA, USA). Then membranes were blocked in 5% non-fat milk for 1 h at room temperature, and incubated with specific antibodies at 4 °C overnight followed by HRP-conjugated secondary antibodies (Jackson Immuno Research, West Grove, PA, USA) at room temperature for one hour. Signals were detected with SuperSignal WestPico Chemiluminescent Substrate (Thermo scientific, Waltham, MA, USA). Each experiment was performed independently at least three times.

### Real-time quantitative PCR (qRT-PCR) analysis

qRT-PCR experiments were performed using the methods described in previous studies. To examine expression of MINCR, total RNAs were collected from NSCLC cells 48 h post transfection, and isolated using Trizol Reagent. First strand cDNA synthesis kit and AceQ qPCR kit were all from Vazyme (Vazyme Biotech, Nanjing, China). The following primer pairs were used: MINCR (Forward: 5′- GTGTCTGGACACCAGAGGAGT − 3′; Reverse: 5′- GGGGCAGAGTCACAAAGC − 3′) and GAPDH: (Forward: 5′- CAAGGTCATCCATGACAACTTTG − 3′; Reverse: 5′- GTCCACCACCCTGTTGCTGTAG -3′) [13, 15, 19, 21].

### Cell cycle and apoptosis analysis

Cells were cultured in 6-cm plates at 1× 10^6^ cells/dish and cultured for 72 h. Silencing the MINCR to measure cell cycle and apoptosis requires siRNA reverse transfection while seeding cells. Cells were collected and subjected to cell cycle and apoptosis assays as described in several previous studies [13, 18, 19]. For cell cycle analysis, cells were collected and incubated with prechilled 70% ethanol overnight at 4 °C. Then, cells were washed with prechilled PBS and incubated with propidium iodide (Sigma-Aldrich, St. Louis, MO, USA) at 37 °C for 1 h. For Apoptosis analysis, cells were collected and incubated with Annexin V-FITC and PI Staining Solution (Yeasen Biotech, Shanghai, China) at room temperature for 30 min in the dark. Both were detected by BD Flow Cytometer (Franklin Lake, NJ, USA), and analyzed using the FlowJo software (Ashland).

### Statistical analysis

All Data are reported as mean ± standard deviation (SD), unless otherwise specified. Significance was analyzed by student’s t test using GraphPad Prism version 5.00 (GraphPad, San Diego, CA, USA) (* *p* < 0.05; ** *p* < 0.01; *** *p* < 0.001; **** *p* < 0.0001) unless otherwise specified.

## Results

### MINCR expression was increased in NSCLC tissues and cell lines

**Fig. 1:**
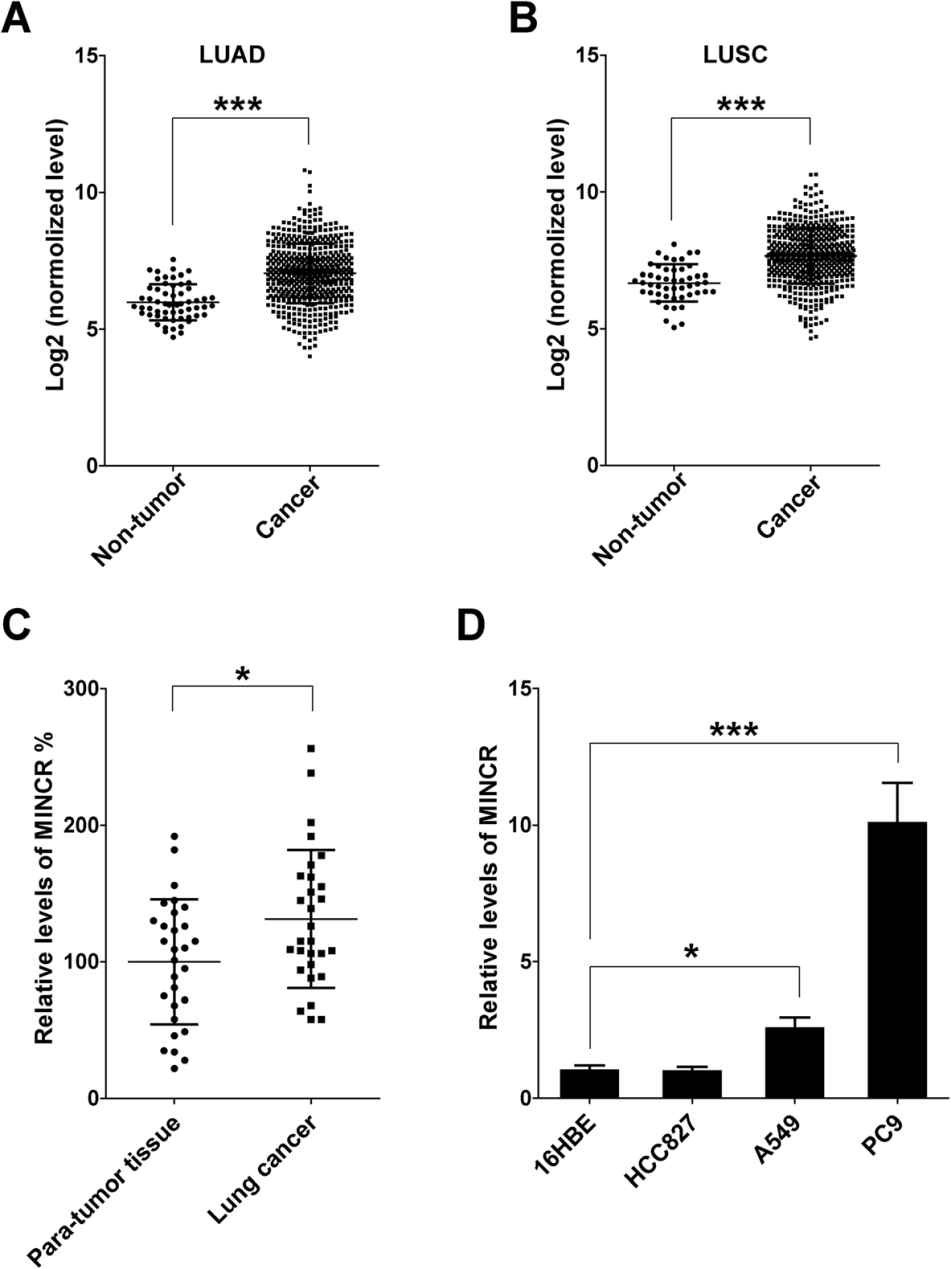
The expression of MINCR in NSCLC tissues and lung cancer cell lines. **a**-**b**, comparing expression of MINCR in non-tumor and NSCLC tissues (Lung Adenocarcinoma, LUAD; Lung Squamous Cell Carcinoma, LUSC) using data from The Cancer Genome Atlas (TCGA). **c**, relative expression levels of MINCR in para-tumor and cancer tissues from NSCLC patients (*N* = 29). **d**, relative expression levels of MINCR in 16HBE, HCC827, A549 and PC9 cell lines (*N* = 3). Data were presented as mean **±** standard deviation (SD); *, *p* < 0.05; **, *p* < 0.01; ***, *p* < 0.001

The expression of MINCR in clinical lung cancers, including LUAD and LUSC, was first identified using TCGA dataset. Results showed that MINCR expression was significantly increased in LUAD and LUSC samples compared with non-tumor controls (Fig. 1a and b). Furthermore, MINCR expression in the lung cancer and para-tumor tissues from 29 NSCLC patients was examined. As shown in Fig. 1c, MINCR expression was significantly increased in cancer tissues compared with para-tumor tissues. Next, the expression of MINCR in NSCLC cell lines (HCC827, A549, PC9) and normal control cell line 16 HBE were detected. As expected, MINCR expression was highly expressed in two NSCLC cell lines, PC9 and A549, especially in PC9 (Fig. 1d). Then, PC9 and A549 were used in further studies.

### Silencing of MINCR significantly inhibited the proliferation of NSCLC cell lines

**Fig. 2:**
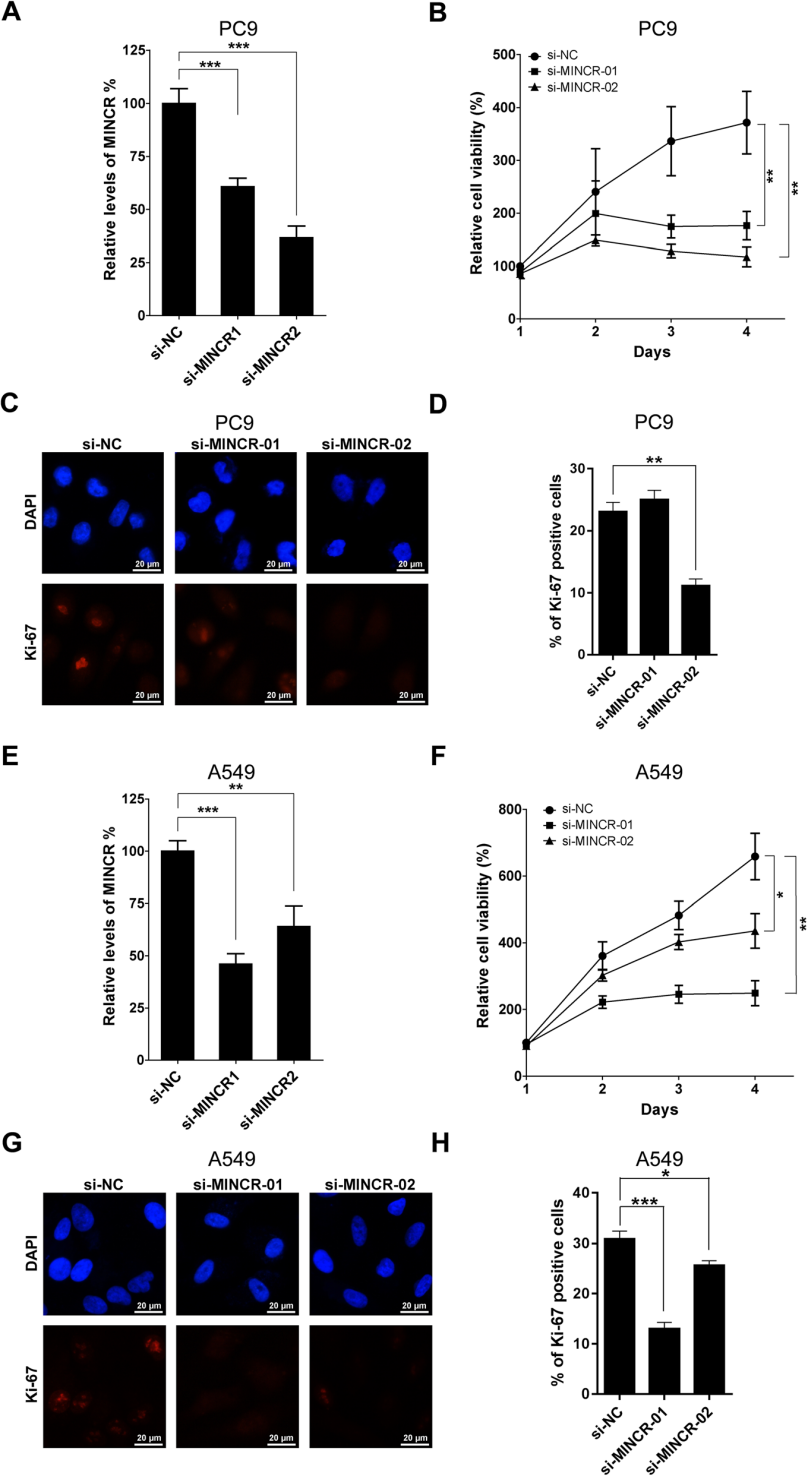
The effect of MINCR silencing on NSCLC cells proliferation. NSCLC cells were transfected with negative control small interfering RNA (siRNA) (si-NC), MINCR siRNA-1 (si-MINCR-01), or MINCR siRNA-2 (si-MINCR-02), respectively, and the viabilities and proliferation were detected using MTT and immunofluorescence. **a-b**, silencing of MINCR in PC9 cells (**a**) and A549 cells (**b**) using siRNAs. **c-e**, The corresponding cell viabilities of PC9 cells (**c**), representative images (**d**) and statistic graphs (**e**) of proliferation marker Ki-67 positive cells. **f-h**, The corresponding cell viabilities of A549 cells (**f**), representative images (**g**) and statistic graphs (**h**) of proliferation marker Ki-67 positive cells. Each experiment was repeated independently at least three times. Data were presented as mean **±** SD; *, *p* < 0.05; **, *p* < 0.01; ***, *p* < 0.001

SiRNAs targeting MINCR, including si-MINCR-01 and si-MINCR-02, were used to silence the expression of MINCR in PC9 and A549 cells. As shown in Fig. 2a, MINCR siRNAs efficiently decreased the expression of MINCR in PC9 cells compared with negative control siRNA. Furthermore, silencing of MINCR greatly inhibited cell viabilities and reduced the expression levels of Ki-67 (Fig. 2b-d), a biomarker for proliferation. Similar results were shown in MINCR-silenced A549 cells (Fig. 2e-h). These data indicated that silencing of MINCR suppressed the proliferation of NSCLC cells.

### Silencing of MINCR inhibited the proliferation of PC9 cells through inhibition of cell cycle and induction of apoptosis

**Fig. 3:**
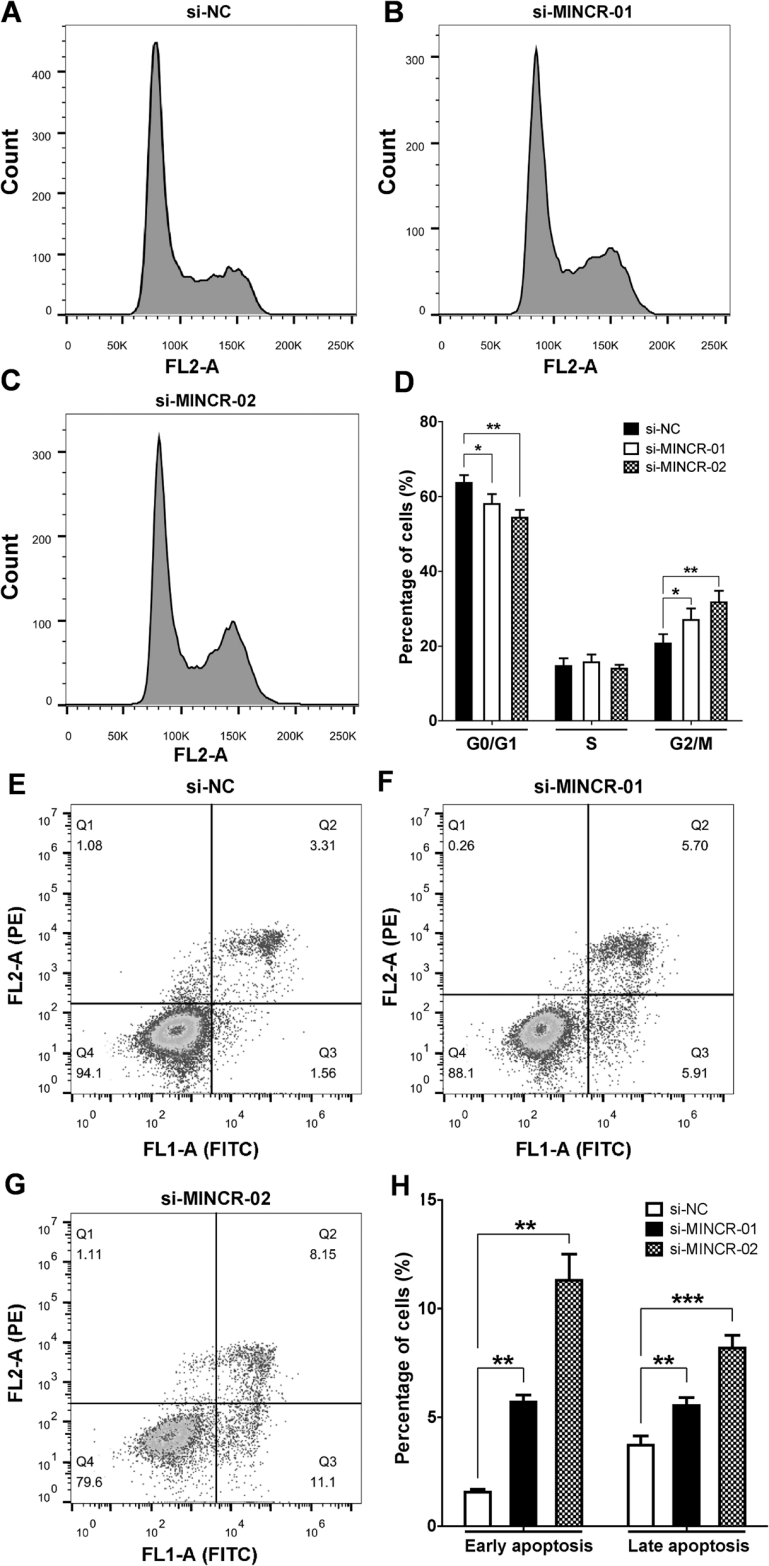
The effect of MINCR silencing on the cell cycle distribution and cell apoptosis of PC9 cells. **a-c** the representative figures of PC9 cells transfected with si-NC, si-MINCR-01, or si-MINCR-02, respectively. **d**, the statistics of experiments (**a-c**). **e-g** the representative figures of PC9 cells transfected with si-NC, si-MINCR-01, and si-MINCR-02, respectively. **h**, the statistics of experiments (**e-g**). Each experiment was repeated independently at least three times. Data were presented as mean **±** SD; *, *p* < 0.05; **, *p* < 0.01; ***, *p* < 0.001

To further dissect the underlying mechanisms by which MINCR silencing inhibited growth of PC9 cells, flow-cytometer was used to analyze the proportions of cells in different cell cycle stages as well as the status of cell appoptosis. As shown in Fig. 3a-d, MINCR silencing significantly decreased G1/0 proportion and increased G2/M proportion of PC9 cells. In addition, MINCR silencing in PC9 cells significantly induced apoptosis in these cells (Fig. 3e-h).

### Silencing of MINCR induced cell cycle arrest and apoptosis of NSCLC cells through decreasing c-Myc expression

**Fig. 4:**
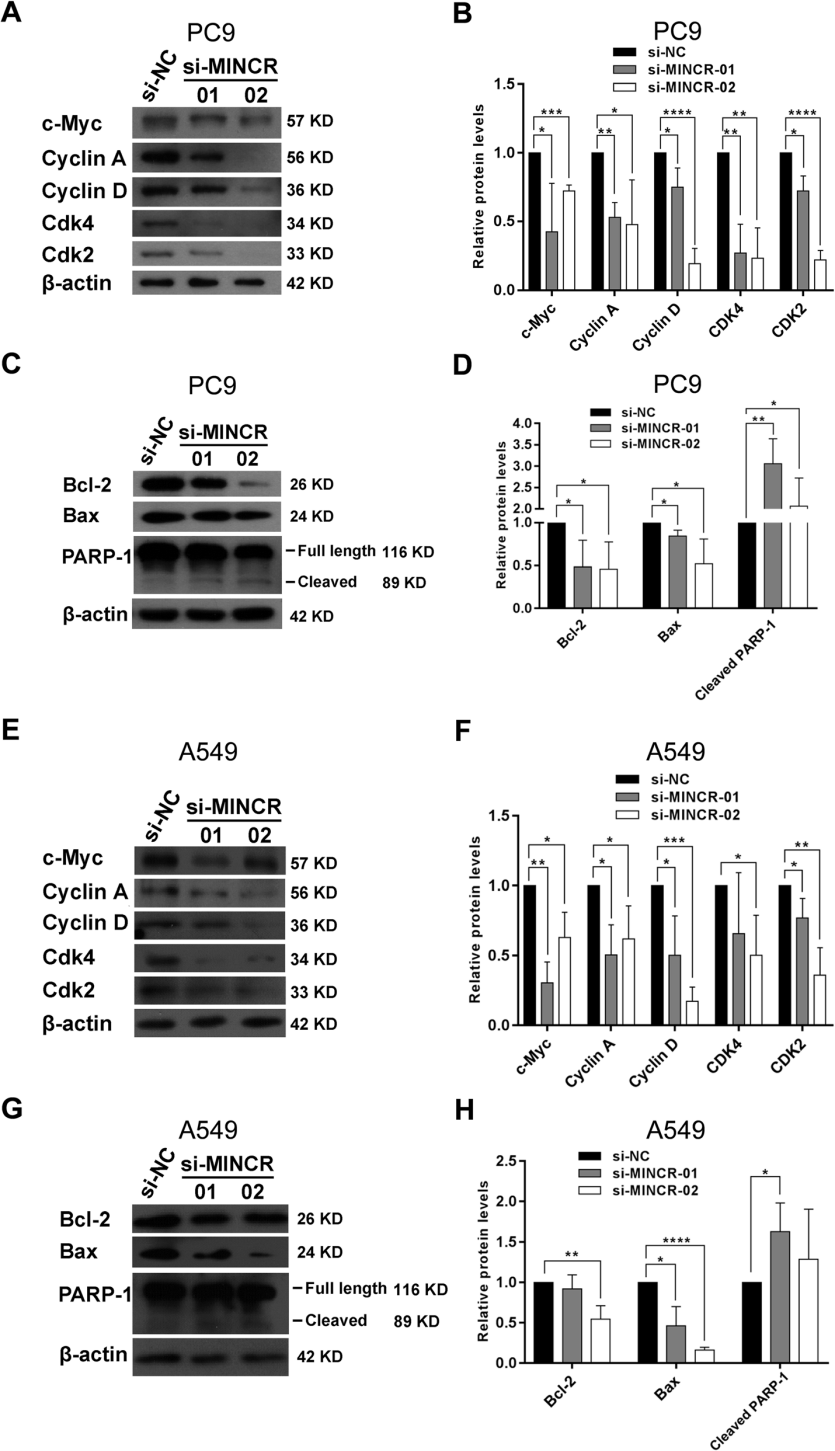
The effects of MINCR silencing on c-Myc and its downstream effectors. **a**-**b**, The protein expression levels (**a**) and statistic graphs (**b**) of c-Myc and its downstream effectors Cyclin A, Cyclin D, CDK4, and CDK2 in PC9 cells with or without MINCR silencing. **c-d**, The protein expression levels (**c**) and statistic graphs (**d**) of Bcl-2, Bax and PARP-1 (full length and cleaved forms) in PC9 cells with or without MINCR silencing. **e-f**, The protein expression levels (**e**) and statistic graphs (**f**) of c-Myc and its downstream effectors Cyclin A, Cyclin D, CDK4, and CDK2 in A549 cells with or without MINCR silencing. **g-h**, The protein expression levels (**g**) and statistic graphs (**h**) of Bcl-2, Bax and PARP-1 (full length and cleaved forms) in A549 cells with or without MINCR silencing. Each experiment was repeated independently at least three times. Data were presented as mean **±** SD; *, *p* < 0.05; **, *p* < 0.01; ***, *p* < 0.001; ****, *p* < 0.0001

Since MINCR is a MYC-induced lncRNA, and has been reported to modulate c-Myc’s transcriptional network in Burkitt lymphoma cells, we sought to determine whether the function of MINCR may associated with the expression of c-Myc and its downstream genes [13]. Results showed that in PC9 cells treated with MINCR siRNA, c-Myc expression was greatly decreased (Fig. 4a). Consistent with this, the expression of several down-stream targets, including cyclin A, cyclin D, CDK2 and CDK4, was significantly decreased after MINCR silencing (Fig. 4a and b). In addition, the expression of cell apoptosis-associated proteins also changed. As expected, the expression of Bcl-2 and Bax, two anti-apoptotic proteins, was decreased, while the expression of cleaved-PARP-1 seemed to be increased, in MINCR-silenced PC9 cells (Fig. 4c and d). In MINCR-silenced A549 cells, expression of c-Myc and proteins associated with cell cycle and apoptosis were also significantly changed (Fig. 4e-h).

### Over-expression of MINCR promoted the proliferation of PC9 cells through increasing c-Myc expression

**Fig. 5:**
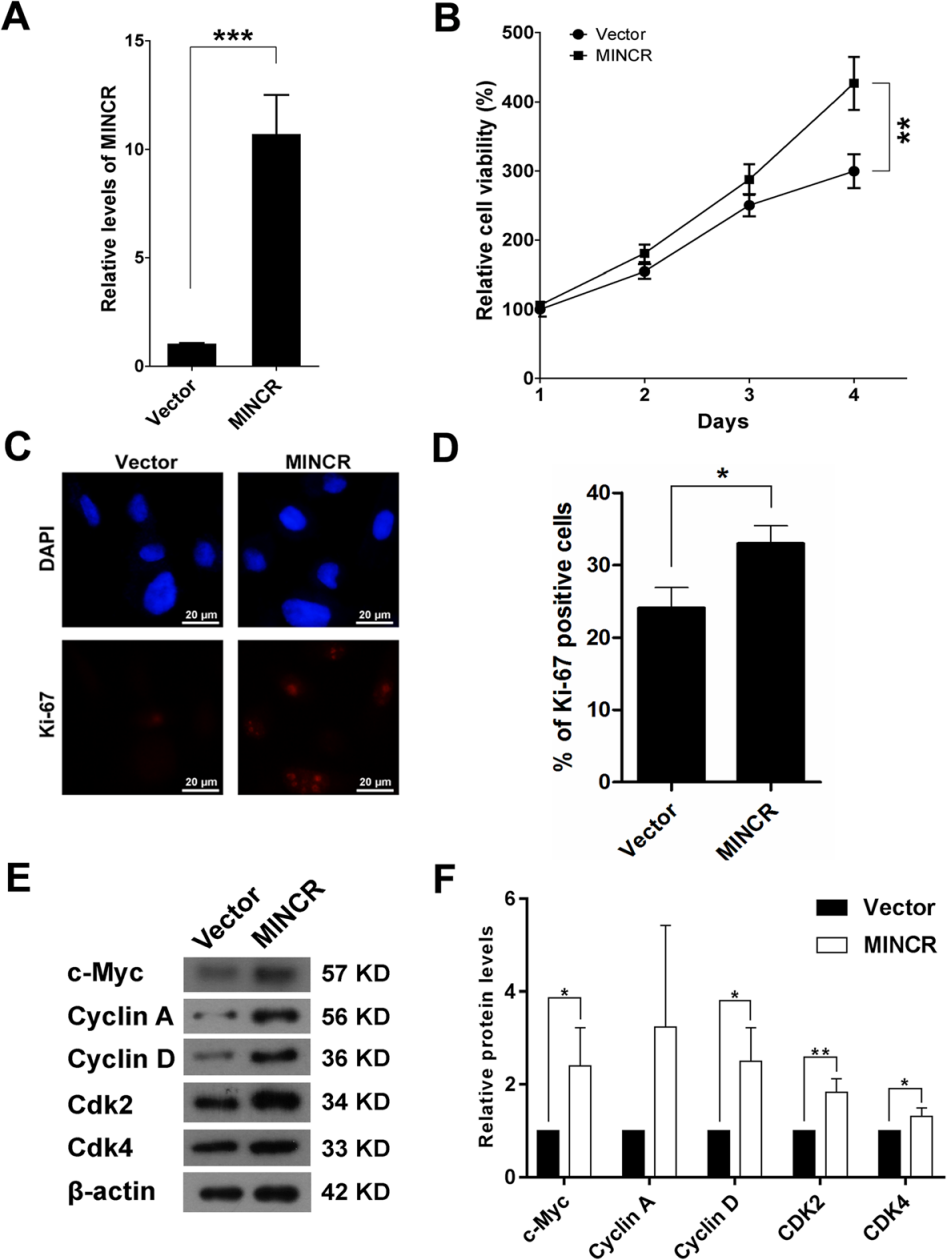
The effect of MINCR over-expression on the proliferation of PC9 cells. **a**,Over-expression of MINCR in PC9 cells using MINCR over-expression vector. **b-d**, the corresponding cell viabilities of PC9 cells (**b**), representative images (**c**) and statistic graphs (**d**) of proliferation marker Ki-67 positive cells. **e-f**, The protein expression levels (**e**) and statistic graphs (**f**) of c-Myc and its downstream effectors Cyclin A, Cyclin D, CDK4, and CDK2 in PC9 cells with or without MINCR over-expression. Each experiment was repeated independently at least three times. Data were presented as mean **±** SD; *, *p* < 0.05; **, *p* < 0.01; ***, *p* < 0.001

To increase the expression of MINCR, MINCR over-expression vector was constructed and then transfected into PC9 cells (Fig. 5a). Results showed that over-expression of MINCR dramatically increased the viabilities and Ki-67 expression (Fig. 5b-d). Mechanism studies showed that in PC9 cells, over-expression of MINCR enhanced c-Myc expression, which in turn increased the expression of some c-Myc downstream effectors, including CDK2, CDK4, Cyclin A, Cyclin D, resulting in promotion of cell cycle progression (Fig. 5e and f). However, in A549 and 16HBE cells, although transfection of MINCR over-expression vector significantly increased the expression of MINCR, the viabilities of these cells were not changed (Additional file 1: Figure S1).

## Discussion

Recent studies have showed that lncRNA MINCR plays oncogenic roles in cancers, such as gallbladder cancer and hepatocellular carcinoma [14, 15]. However, its precise roles in NSCLC and the underlying mechanisms are not fully elucidated. In this study, we identified that MINCR was highly increased in NSCLC tissues and cell lines. Furthermore, silencing of MINCR greatly reduced cell growth by inducing cell cycle arrest and apoptosis of NSCLC cells, while over-expressing MINCR remarkably enhanced cell proliferation, part of which are consistent with a previous report by Wang et al. [24]. For the mechanism studies, Wang et al. suggested that MINCR/ miR-126/ solute carrier family 7 member 5 (SLC7A5) axis was involved in NSCLC progression [24]. Interestingly, our study elucidated a positive regulation of MINCR on c-Myc and its target genes, indicating a positive auto-regulatory loop between MINCR and c-Myc allowing their higher expression in NSCLC and accelerating NSCLC progression. Thus, MINCR may serve as a promising therapeutic target for the treatment of NSCLC.

C-Myc, a classical transcription factor, has been reported to regulate the expression of 15% of all human protein-coding genes, as well as some non-coding genes [25, 26]. It was found to be greatly increased in different human cancers, and played pivotal roles in cancer progression [27]. Since MINCR was reported to be a modulator of the c-Myc transcriptional network in Burkitt lymphoma cells, we postulated that the function of MINCR in NSCLC may be associated with its regulation of c-Myc expression [13]. As expected, we found that silencing of MINCR reduced the expression of c-Myc and its target effectors, while over-expression of MINCR activated the expression of c-Myc and its target genes, indicating that MINCR was capable of promoting c-Myc transcription network in NSCLC cells. Further studies should explore the underlying mechanism of the regulation of c-Myc by MINCR in NSCLC.

Currently, several lncRNAs were reported to regulate c-Myc at multiple levels, including transcription, translation, protein stability and activity, by interacting with different partners. For example, lncRNA colon cancer associated transcript 2 (CCAT2) promotes c-Myc transcription by strengthenning the binding of transcription factor transcription factor 7 like 2 (TCF7L2) to the c-Myc promoter in colon cancer; testis-associated highly-conserved oncogenic lncRNA (THOR) enhances c-Myc mRNA stability by interacting with insulin like growth factor 2 mRNA binding proteins (IGF2BPs) and facilitating its binding to c-Myc mRNA in skin squamous cell carcinoma; lncRNA prostate cancer associated transcript 1 (PCAT-1) stimulates c-Myc mRNA translation by competitively interacting with the 3′-UTR of c-Myc mRNA and prevent its binding with miR-34a-5p; whereas lncRNA protein disulfide isomerase family A member 3 pseudogene (PDIA3P) promotes c-Myc activities by increasing the occupancy of c-Myc at the promoter of its target gene glucose-6-phosphate dehydrogenase (G6PD) in multiple myeloma cells [28–31]. In our current study, accompanied with the modulation of MINCR on c-Myc, the expression of c-Myc target genes exihibited the same trend, which is similar with the results from Doose’s studies in Burkit lymphoma cells [13]. In following investigations, the direct or indirect interaction between MINCR and c-Myc needs to be studied, and whether MINCR promotes c-Myc by enhancing the binding of c-Myc to the promoters of its target effectors need to be determined. Besides, the relationship of MINCR and c-Myc need to be further assured in vivo.

## Conclusions

Taken together, the current study indicated that MINCR as a regulator in NSCLC to enhance NSCLC progression by effectively inhibiting cell cycle arrest and apoptosis via increasing expression of c-Myc and its target effectors, which sheds light on the possible application of silencing of MINCR for the treatment of NSCLC.

## Additional file

Additional file 1: **Figure S1.** The effect of MINCR over-expression on the viability of A549 and 16HBE cells. **(A-B)**, Over-expression of MINCR in A549 cells using MINCR over-expression vector **(A)**, and the corresponding cell viabilities of A549 cells **(B)**. **(C-D)**, Over-expression of MINCR in 16HBE cells using MINCR over-expression vector **(C)**, and the corresponding cell viabilities of A549 cells **(D)**. Each experiment was repeated independently at least three times. Data were presented as mean **±** SD; ***, *p* < 0.001. (TIF 3238 kb)

## Data Availability

All data generated or analyzed during this study are available from the corresponding author on reasonable request.

## Abbreviations

CDK: cylin-dependent kinase
EZH2: enhancer of zeste homolog 2
lncRNA: Long non-coding RNA
LUAD: lung adenocarcinoma
LUSC: lung squamous carcinoma
MINCR: MYC-induced long non-coding RNA
NSCLC: Non-small cell lung cancer
siRNA: small interfering RNA
TCGA: The Cancer Genome Atlas
