# Supplementary figures and images for: Roles of MYC-targeting long non-coding RNA MINCR in cell cycle regulation and apoptosis in non-small cell lung Cancer
Source: Respir Res. 2019 Sep 3;20:202. doi: 10.1186/s12931-019-1174-z (PMC6724276; doi:10.1186/s12931-019-1174-z)

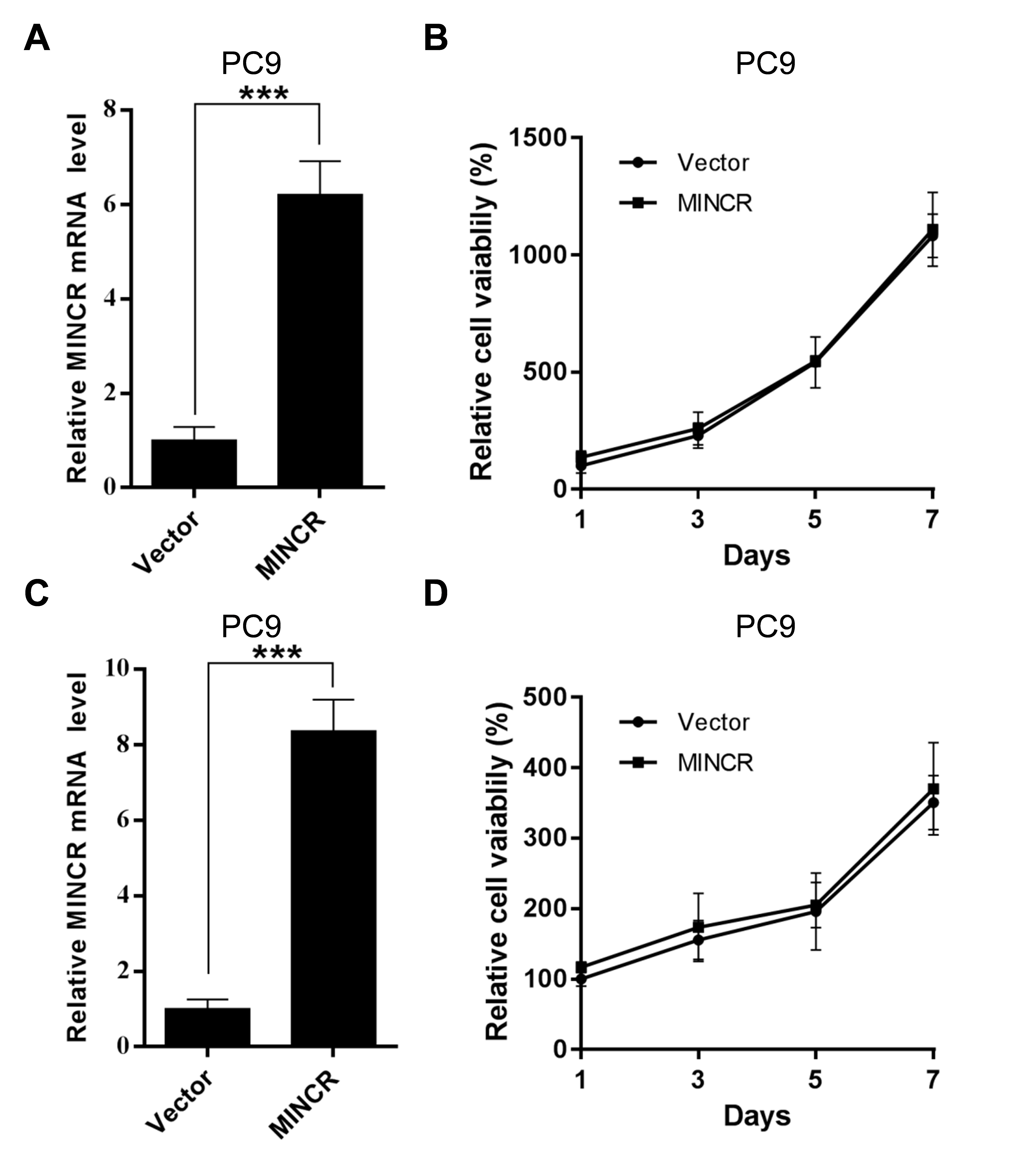

Supplement: Supplementary file 1 — Figure S1. The effect of MINCR over-expression on the viability of A549 and 16HBE cells. (A-B), Over-expression of MINCR in A549 cells using MINCR over-expression vector (A), and the corresponding cell viabilities of A549 cells (B). (C-D), Over-expression of MINCR in 16HBE cells using MINCR over-expression vector (C), and the corresponding cell viabilities of A549 cells (D). Each experiment was repeated independently at least three times. Data were presented as mean ± SD; ***, p < 0.001. (TIF 3238 kb) [file 12931_2019_1174_MOESM1_ESM.tif]
